# Acetyl-CoA-carboxylase 1 (ACC1) plays a critical role in glucagon secretion

**DOI:** 10.1038/s42003-022-03170-w

**Published:** 2022-03-18

**Authors:** Anna Veprik, Geoffrey Denwood, Dong Liu, Rula Bany Bakar, Valentin Morfin, Kara McHugh, Nchimunya N. Tebeka, Laurène Vetterli, Ekaterina Yonova-Doing, Fiona Gribble, Frank Reimann, Kyle L. Hoehn, Piers A. Hemsley, Jonas Ahnfelt-Rønne, Patrik Rorsman, Quan Zhang, Heidi de Wet, James Cantley

**Affiliations:** 1 Department of Physiology, Anatomy and Genetics, University of Oxford, Oxford, UK; 2 Novo Nordisk Research Centre Oxford, Oxford, UK; 3 The Oxford Centre for Diabetes, Endocrinology and Metabolism, University of Oxford, Oxford, UK; 4 Division of Systems Medicine, School of Medicine, University of Dundee, Dundee, Scotland UK; 5 School of Life Sciences, University of Dundee, Dundee, Scotland UK; 6 Metabolic Research Laboratories and MRC Metabolic Diseases Unit, Institute of Metabolic Science, Addenbrooke’s Hospital, Cambridge, UK; 7 School of Biotechnology and Biomolecular Sciences, University of New South Wales, Sydney, NSW Australia; 8 Cell and Molecular Sciences, The James Hutton Institute, Dundee, Scotland UK; 9 Department of Pathology and Imaging, Novo Nordisk A/S, Måløv, Denmark

**Keywords:** Homeostasis, Type 2 diabetes, Nutrient signalling

## Abstract

Dysregulated glucagon secretion from pancreatic alpha-cells is a key feature of type-1 and type-2 diabetes (T1D and T2D), yet our mechanistic understanding of alpha-cell function is underdeveloped relative to insulin-secreting beta-cells. Here we show that the enzyme acetyl-CoA-carboxylase 1 (ACC1), which couples glucose metabolism to lipogenesis, plays a key role in the regulation of glucagon secretion. Pharmacological inhibition of ACC1 in mouse islets or αTC9 cells impaired glucagon secretion at low glucose (1 mmol/l). Likewise, deletion of ACC1 in alpha-cells in mice reduced glucagon secretion at low glucose in isolated islets, and in response to fasting or insulin-induced hypoglycaemia in vivo. Electrophysiological recordings identified impaired K_ATP_ channel activity and P/Q- and L-type calcium currents in alpha-cells lacking ACC1, explaining the loss of glucose-sensing. ACC-dependent alterations in S-acylation of the K_ATP_ channel subunit, Kir6.2, were identified by acyl-biotin exchange assays. Histological analysis identified that loss of ACC1 caused a reduction in alpha-cell area of the pancreas, glucagon content and individual alpha-cell size, further impairing secretory capacity. Loss of ACC1 also reduced the release of glucagon-like peptide 1 (GLP-1) in primary gastrointestinal crypts. Together, these data reveal a role for the ACC1-coupled pathway in proglucagon-expressing nutrient-responsive endocrine cell function and systemic glucose homeostasis.

## Abstract (web summary)

Veprik et al. show that Acetyl-CoA-carboxylase 1 (ACC1), an enzyme that couples glucose metabolism to lipogenesis, is involved in glucagon secretion and regulates S-acylation of critical glucose-sensing proteins. Loss of ACC1 in pancreatic alpha-cells negatively affects both size and number, as well as glucagon content, while in gut enteroendocrine cells leads to reduced release of glucagon-like peptide 1.

## Introduction

Regulated insulin and glucagon secretion from pancreatic islets maintains metabolic homoeostasis. Glucagon acts on multiple tissues including the liver, where it promotes hepatic glucose production to raise blood glucose concentrations, counter-regulatory to the glucose-lowering actions of insulin. As such, glucagon secretion is enhanced at low glucose (peaking at ~1 mmol/l) and suppressed at high glucose concentrations^1^, reciprocal to glucose-stimulated insulin secretion from pancreatic beta-cells. T2D is not only characterised by hyperglycaemia, insulin resistance and pancreatic beta-cell dysfunction^2^, but also by excessive glucagon release at fasting and postprandial glucose concentrations, exacerbating hyperglycaemia^3–6^. Furthermore, impaired glucagon secretion in response to hypoglycaemia occurs in both T1D and T2D^5^.

The contribution of glucagon to hyperglycaemia has been underscored by several recent studies: glucagon receptor deletion prevented diabetes from streptozotocin-induced insulin-deficiency in mice^7^, whilst glucagon receptor antagonism reduced blood glucose in insulin-resistant mice^8^ and humans with T2D^9^. As glucagon plays a key role in metabolic homoeostasis and disease, deeper insights into alpha-cell function are needed to better understand dysregulated glucagon secretion and—ultimately—to enable new therapeutic strategies targeting the alpha-cell. In the present study, we reveal a critical role for ACC1 in the intrinsic regulation of glucagon secretion using a range of in vivo, ex vivo and in vitro models. Pharmacological or genetic inhibition of ACC1 resulted in impaired glucagon secretion at low glucose and in response to fasting or insulin-induced hypoglycaemia in vivo. We used a combination of secretion experiments and electrophysiological recordings to show that loss of ACC1 in alpha cells restricts K_ATP_ channel activity at low glucose, with reduced P/Q- and L-type calcium currents, revealing the mechanistic defects underpinning the loss of glucose sensing. Our study further reveals a role for ACC1 activity in maintaining alpha cell volume and glucagon content and a role for ACC1 in GLP-1 secretion from primary gastrointestinal crypts.

## Results

### ACC1 is expressed in pancreatic alpha cells

Acetyl-CoA carboxylase 1 (ACC1/ACACA) couples anaplerotic glucose metabolism with de novo lipogenesis, by forming malonyl-CoA from cytosolic acetyl-CoA^10^. In beta-cells, this pathway plays a critical role in regulating insulin secretion^11–15^. Transcriptomic data show that the ACC1 gene (*Acaca*) is expressed at similar levels in alpha- and beta-cells^16–19^. We, therefore, hypothesised that ACC1 may contribute to the intrinsic glucose-regulation of glucagon secretion. We first determined ACC1 expression in a range of tissues at the mRNA and protein level (Supplementary Fig. 1a, b): ACC1 was detected in all tissues examined, and was highly expressed in liver, a lipogenic tissue. Importantly, ACC1 expression was detected in primary mouse islets and the transformed mouse αTC9 alpha-cell line^20,21^. We used a label-free fluorescence-activated cell sorting (FACS) protocol to enrich primary alpha-cells from dissociated islets, with alpha-cell purity confirmed by reverse transcription-polymerase chain reaction (RT-PCR) (Supplementary Fig. 1c–f). ACC1 expression was detected at comparable levels in primary alpha and beta-cell populations (Supplementary Fig. 1a). Notably, ACC1 (*Acaca*) mRNA expression was ~18-fold higher than ACC2 (*Acacb*) in alpha-cells, and ~121-fold higher than ACC2 in beta-cells, indicating that ACC1 is the principal ACC isoform in these cell types, consistent with previous data^16–19^.

### Inhibition of ACC1 impairs glucagon secretion

**Fig. 1:**
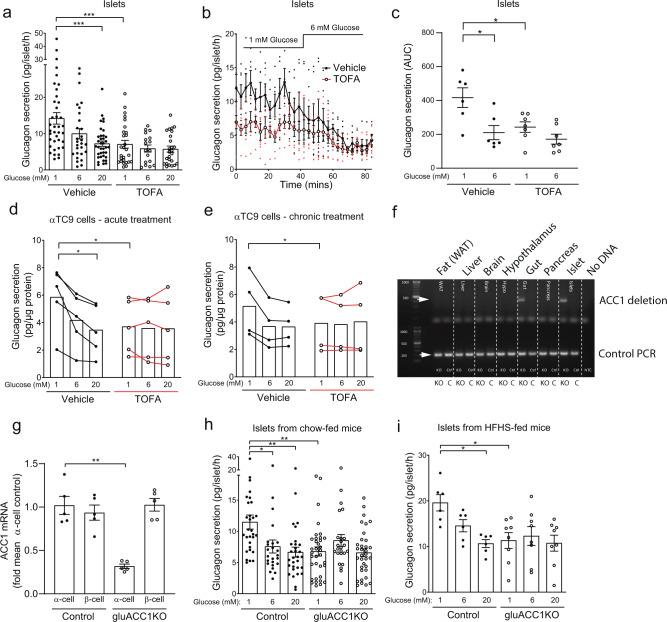
Inhibition or genetic inactivation of ACC1 impairs glucagon secretion. **a** Pancreatic islets isolated from wild-type C57BL6/J mice (10–16 weeks of age) were treated with 25 µM TOFA (ACC inhibitor; white circles) or vehicle (black circles) for 1 h, prior to glucagon secretion assays. Data from *n* = 24–34 independent islet preparations were analysed by one-way ANOVA with Tukey multiple-comparisons test. **b**, **c** Wild-type islets treated with vehicle or TOFA for 1 h prior to the assessment of time-resolved glucagon secretion by perifusion. Mean trace data (**b**) and area under the curve (**c**) are presented. Data from *n* = 6–7 independent islet preparations analysed by *t*-test. **d**, **e** αTC9 cells treated with TOFA for 1 h (**d**) or 24 h (**e**) prior to glucagon secretion experiments, with secretion data normalised to protein content per well. Data from *n* = 4–5 independent experiments analysed by paired *t* test. **f** Confirmation of ACC1 gene deletion in pancreatic alpha-cells. Genomic DNA was isolated from the indicated tissues and amplified using primers spanning the deleted region or a control region, to verify tissue specificity of ACC1 (*Acaca*) deletion. Gel represents three independent experiments analysing three independent mice. **g** ACC1 mRNA expression assessed by RT-PCR using cDNA from FACS-enriched alpha and beta cell populations, from *n* = 5 independent islet preparations analysed by Mann–Whitney test. **h** Pancreatic islets isolated from adult control (black circles) and gluACC1KO (white circles) mice, and glucagon secretion assessed. Data from *n* = 24–35 independent islet preparations were analysed by one-way ANOVA with Tukey’s multiple-comparisons test. **i** Glucagon secretion was assessed from islets isolated from mice following 12 weeks of exposure to a high-fat high-sucrose diet. Data from *n* = 6–8 independent islet preparations were analysed by one-way ANOVA with Tukey’s multiple-comparisons test. Data presented as mean ± SEM. Significance thresholds: *P < 0.05, **P < 0.01, ***P < 0.001.

To investigate the role of ACC1 in alpha-cell function, we utilised the small-molecule ACC1 inhibitor 5-(tetradecyloxy)-2-furoic acid (TOFA), which has previously been used to inhibit ACC1 in beta cells^13^. Treatment of isolated mouse islets with TOFA (25 µmol/l) rendered glucagon secretion insensitive to glucose, with a significant impairment at 1 mmol/l glucose (Fig. 1a). Likewise, during dynamic secretion assays (perifusion), glucagon release at 1 mmol/l glucose was impaired in TOFA-treated islets, resulting in a flattened glucose response (Fig. 1b, c). To test the cell-intrinsic role of ACC1 independently of islet paracrine factors, we utilised the transformed αTC9 alpha-cell line. Both acute (Fig. 1d) and chronic (Fig. 1e) TOFA-treatment of αTC9-cells resulted in a significant reduction of glucagon release at 1 mmol/l glucose, closely mirroring the pattern of glucagon secretion in TOFA-treated primary islets (Fig. 1a). These in vitro data support a role for intrinsic ACC1 activity in glucose-regulated glucagon secretion.

### Genetic ablation of ACC1 in alpha cells impairs glucagon release ex vivo

We next sought to investigate the role of ACC1 in alpha-cell function by deleting the *Acaca* gene in proglucagon-expressing cells. Transgenic mice expressing Cre-recombinase under the control of the glucagon promoter^22^ were crossed with ACC1-floxed mice^23^ to generate gluACC1KO and littermate ACC1-floxed control mice. Deletion of the floxed ACC1 allele was only detected in pancreatic islets and gut explants from gluACC1KO mice (Fig. 1f). No deletion was detected in any tissue from ACC1-floxed control mice. Alpha cell ACC1 deletion was further confirmed using RT-PCR of mRNA extracted from FACS enriched alpha and beta-cell populations (Fig. 1g).

To assess the impact of ACC1 deletion on the alpha-cell function we isolated pancreatic islets from gluACC1KO and control mice and performed static glucagon secretion experiments ex vivo. Lowering glucose concentrations (to 1 mmol/l) in control islets stimulated glucagon secretion. In contrast, glucagon secretion from gluACC1KO islets was insensitive to changes in glucose concentrations, with significantly less glucagon released at 1 mmol/l glucose relative to controls (Fig. 1h, Supplementary Fig. 2a). This lack of glucose-sensing observed in the KO islets phenocopies the impact of short-term ACC1 inhibition in both islets and αTC9-cells, reinforcing the role of ACC1 in regulating glucagon secretion. Insulin secretion from gluACC1KO islets was unaltered both under basal and glucose-stimulated conditions (Supplementary Fig. 2b), demonstrating that beta-cell function is unaffected by deletion of ACC1 in neighbouring alpha-cells.

To test if ACC1 plays a role in alpha-cell adaptation to obesity, mice were fed a high-fat high-sucrose (HFHS) diet for 12 weeks. Glucagon secretion from islets isolated from HFHS-diet-fed mice of both genotypes was ~1.7-fold higher relative to islets from chow-fed mice (Fig. 1h, i) (islet glucagon secretion pg/islet/h at 1mmol/l glucose: chow-fed control 11.48 ± 1.50 vs. HFHS-fed control 19.58 ± 1.55, *P* = 0.0045; chow-fed gluACC1KO 6.76 ± 1.05 vs. HFHS-fed gluACC1KO 11.31 ± 1.72, *P* = 0.0123; mean ± SEM, *t*-test), demonstrating that gluACC1KO mice have the same ability as control mice to increase overall glucagon secretion in response to a chronic dietary challenge and associated obesity. However, notwithstanding this augmentation of tonic glucagon secretion, gluACC1KO alpha cells remained insensitive to glucose concentrations in islets isolated from HFHS-diet-fed mice (Fig. 1i) indicating a persistent glucose-sensing defect.

### Impaired glucose tolerance and fasting glucagon secretion in gluACC1KO mice

**Fig. 2:**
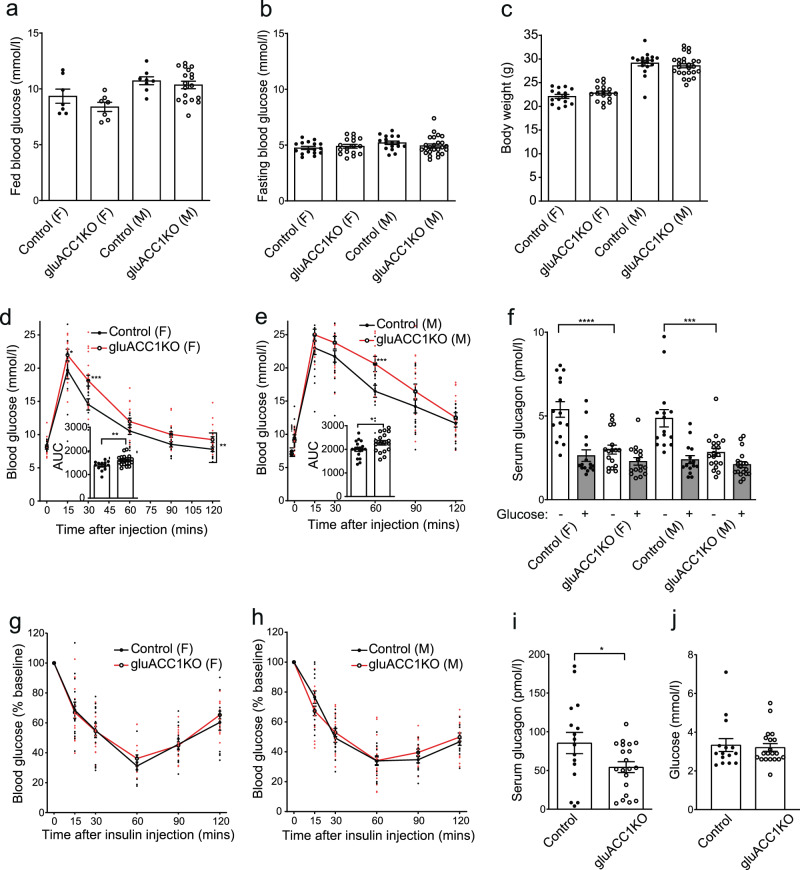
Genetic inactivation of ACC1 in alpha-cells impairs glucagon secretion in vivo. **a**, **b** Blood glucose levels measured in 20–22 week old control (black circles) and gluACC1KO (white circles) mice in the fed (**a**; *n* = 7–18) and 16 h fasted (**b**; *n* = 16–25) states. **c** Body weight measured in 20–22-week-old control (black circles) and gluACC1KO (white circles) mice (*n* = 15–25 mice). **d**, **e** Blood glucose levels and AUC during an IPGTT (6 h fast; 2 g/kg glucose dose) performed in 12–14-week-old female (**d**; *n* = 13–19) and male (**e**; *n* = 18) mice. Trace data analysed by two-way ANOVA (*P* = 0.0009 for genotype effect in (**d**) with Fisher’s LSD test indicated. AUC was analysed by *t*-test. **f** Serum glucagon levels during the IPGTT at time 0 (white bars) and 30 min post-glucose injection (grey bars). Data from *n* = 15–19 mice analysed by *t*-test. **g**, **h** Insulin tolerance test (ITT) performed following 6 h fasting in female mice injected i.p. with 0.5 U/kg insulin (**g**; n = 16-17), or male mice injected i.p. with 0.75 U/kg insulin (**h**; *n* = 16–19). **i**, **j** Serum glucagon (**i**) and blood glucose (**j**) concentrations 60 min post-insulin injection in female mice. Data from *n* = 16–20 mice analysed by *t*-test. Data presented as mean ± SEM, for female (F) and male (M) mice as indicated. Significance thresholds: *P < 0.05, **P < 0.01, ***P < 0.001, ****P < 0.0001.

We next assessed the impact of ACC1 deletion in glucagon-expressing cells on whole-body metabolic homoeostasis. Fed and fasting blood glucose concentrations (Fig. 2a, b), body weight (Fig. 2c) and food intake (Supplementary Fig. 3a) were unaltered in gluACC1KO mice relative to controls. Bodyweight and gonadal fat mass were equivalent between genotypes after 12 weeks on HFHS diet (Supplementary Fig. 3b, c), further supporting the view that loss of ACC1 in proglucagon-expressing cells does not alter body weight regulation. Both female and male gluACC1KO mice exhibited impaired glucose tolerance during an i.p. glucose tolerance test (IPGTT), relative to their respective controls (Fig. 2d, e). As expected, levels of circulating glucagon in control mice were elevated in the fasted state and decreased by ~50% following glucose stimulation (Fig. 2f). In contrast, fasting glucagon levels were significantly lower in gluACC1KO mice relative to controls, and did not change following glucose injection (Fig. 2f), indicating an insensitivity of ACC1-null alpha-cells to the prevailing blood glucose concentration, consistent with our in vitro data. Moreover, lower glucagon levels and normal blood glucose concentrations during fasting suggest that gluACC1KO mice require less glucagon to maintain euglycaemia, and the failure to reduce serum glucagon concentrations (and therefore glucagon action) following glucose injection may contribute to the glucose-intolerant phenotype.

Changes in blood glucose during insulin tolerance tests were comparable between genotypes, indicating normal insulin action in gluACC1KO mice (Fig. 2g, h). Serum glucagon concentrations increased markedly (~16-fold) in both genotypes following insulin-induced hypoglycaemia (Fig. 2i, j) compared to non-insulin treated mice (Fig. 2f). We attribute this augmented secretory response to extrinsic factors, such as adrenaline and the autonomic nervous system, which act to raise blood glucose via glucagon dependent and independent mechanisms^24,25^. Furthermore, noradrenaline treatment of wild-type islets ex vivo enhanced glucagon secretion at low glucose as previously reported^26^, and acute inhibition of ACC1 with TOFA treatment did not diminish this response (Supplementary Fig. 4a), indicating that the adrenaline response remains intact in ACC1 deficient alpha cells. Notwithstanding this, gluACC1KO mice showed significantly lower circulating glucagon levels relative to controls in vivo (Fig. 2i), further demonstrating an impaired capacity for glucagon secretion in ACC1-null alpha-cells, suggesting a reduction in alpha cell mass or glucagon content. Interestingly, despite the severe impairment in glucose sensing and glucagon secretion, this did not result in severe hypoglycaemia in gluACC1KO mice (relative to controls) after fasting (Fig. 2b) or insulin injection (Fig. 2j). This is in agreement with the report that mice with global deletion of the glucagon receptor show normal fasting blood glucose concentrations despite the loss of systemic glucagon action^7^, highlighting the redundancy in glucose homeostatic mechanisms.

### Loss of ACC1 activity in L-cells impairs GLP-1 release

**Fig. 3:**
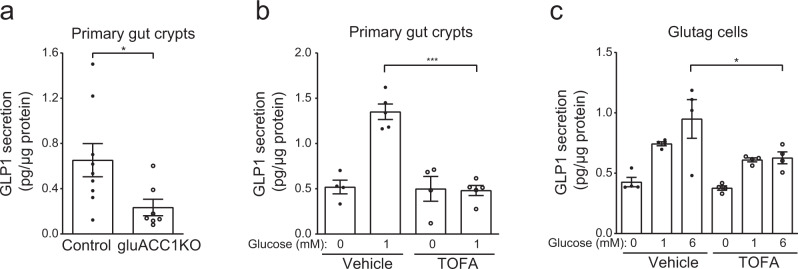
Inactivation of ACC1 impairs glucose-stimulated active GLP-1 secretion. **a** Primary gastrointestinal (GI) crypts were isolated from 20- to 24-week old control (black circles) and gluACC1KO (white circles) mice and secretion of active GLP1 was quantified ex vivo in the presence of 1 mmol/l glucose, and normalised to the total protein content of each well. Data from *n* = 7–9 independent preparations analysed by *t*-test. **b** Active GLP-1 secretion from primary wild-type (C57Bl6/J) GI crypts treated with the ACC inhibitor TOFA (white circles) or vehicle (black circles), and the indicated glucose concentrations. Data from *n* = 5 independent preparations analysed by *t*-test. **c** Active GLP-1 secretion from transformed Glutag cells treated with TOFA or vehicle, in the presence of the indicated glucose concentrations. Data from *n* = 4 independent experiments were analysed by one-way ANOVA with Tukey’s multiple-comparisons test. Data presented as mean ± SEM. Significance thresholds: *P < 0.05, ***P < 0.001.

Although we primarily focus on the role of ACC1 in alpha-cells, our gluACC1KO mouse also results in ACC1 deletion in proglucagon-expressing cells of the gut (Fig. 1f). In enteroendocrine L-cells, proglucagon is processed by prohormone convertase 1/3 to produce GLP-1, which is secreted in response to food intake via stimulation of L-cells by glucose and other nutrients^27,28^. GLP-1 acts on a range of tissues that express the GLP-1 receptor, including potentiating insulin release, reducing gastric motility and promoting post-prandial satiety^29^. As fed blood glucose (Fig. 2a), food intake (Supplementary Fig. 3a), circulating total GLP-1 (Supplementary Fig. 3d) and insulin after oral glucose (Supplementary Fig. 3e) were normal in gluACC1KO mice, this suggests that there is no major alteration in gut GLP-1. Nevertheless, we opted to investigate the role of L-cell ACC1 in the intrinsic regulation of GLP-1 release in vitro. We identified that secretion of active-GLP-1 was reduced by 63% from intestinal crypts isolated from gluACC1KO mice, relative to controls (Fig. 3a). We treated crypts isolated from wild-type mice (Fig. 3b) and the transformed Glutag cell line (Fig. 3c) with TOFA: ACC-inhibition caused a significant reduction in GLP-1 secretion relative to vehicle-treated cells in both models.

### Impaired K_ATP_ channel conductance underpins the secretory defect in gluACC1KO alpha cells

**Fig. 4:**
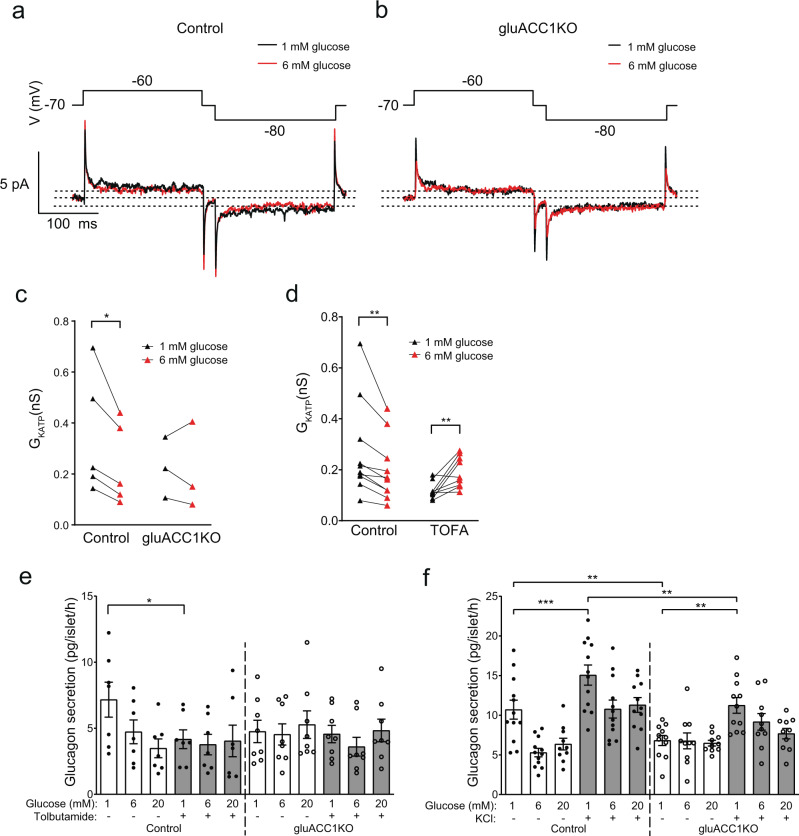
Impaired glucose-regulation of K_ATP_-channels in gluACC1KO alpha-cells. **a**–**c** Electrophysiological recordings of K^+^ currents triggered by ±10 mV excursions (200 ms) from −70 mV in voltage-clamped primary control or gluACC1KO alpha-cells, in the presence of 1 or 6 mmol/l glucose (black or red traces/symbols, respectively). **a**, **b** Representative traces of K^+^ currents. Dotted lines mark steady-state currents at −60, −70 and −80 mV at 6 mmol/l glucose. **c** Summary data presented as K_ATP_ channel conductance (GKATP(nS)) from individual cells from *n* = 3–5 independent preparations analysed by paired *t*-test. **d** Assessment of K_ATP_ channel conductance (as described for **a**–**c**) in primary alpha-cells treated with 0.02% DMSO vehicle (Control) or 25 mmol/l TOFA for 1 h prior to, and during, recordings, to inhibit ACC1 activity. Summary data presented as K_ATP_ channel conductance (GK_ATP_(nS)) from *n* = 9–10 independent preparations analysed by paired *t*-test. **e** Pancreatic islets isolated from control (black circles) and gluACC1KO (white circles) mice were used to quantify glucagon secretion in response to the indicated glucose concentrations, combined with vehicle (white bars) or 200 mmol/l Tolbutamide treatment (grey bars) to inhibit K_ATP_ channel activity. Data from *n* = 7–8 independent islet preparations were analysed by two-way ANOVA with Fisher’s LSD test. **f** Islet glucagon secretion assessed in response to the indicated glucose concentrations, combined with vehicle (white bars) or 70 mmol/l KCl treatment (grey bars). Data from *n* = 10–12 independent islet preparations were analysed by two-way ANOVA with Fisher’s LSD test. All data presented as mean ± SEM. Significance thresholds: *P < 0.05, **P < 0.01, ***P < 0.001.

We considered the possibility that the defective glucose-sensing in gluACC1KO alpha-cells reflects dysregulation of the K_ATP_ channel, which has been proposed to play a key role in alpha cell function^1,30^. To assess this, we used a perforated whole-cell patch–clamp technique and recorded K_ATP_ channel activity (expressed as conductance) at 1 and 6 mmol/l extracellular glucose. In control alpha-cells, K_ATP_ channel conductance is significantly elevated at 1 mmol/l glucose, relative to 6 mmol/l glucose, whereas no significant increase was observed in gluACC1KO alpha-cells (Fig. 4a–c). To determine the impact of acute ACC1 inhibition on K_ATP_ channel conductance we repeated these electrophysiological recordings using primary wild type mouse islets treated with TOFA or vehicle. Again, K_ATP_ channel activity was lower at 6 mM than at 1 mM glucose in controls (Fig. 4d). In contrast, in the presence of TOFA alpha cell K_ATP_ channel activity was low at 1 mmol/l glucose, and showed a small but significant increase with 6 mmol/l glucose rather than decreased K_ATP_ conductance (Fig. 4d).

Alongside K_ATP_ channels, voltage-gated sodium channels (Na_v_ channels) play an important role in alpha cell electrical activity and glucose sensing^1,31^. Therefore, we monitored the impact of ACC1 deletion on alpha cell Na_v_ channel activity (Supplementary Fig. 5). Voltage-dependent Na_v_ inactivation was tested using a standard two-pulse protocol^31^ and the total depolarisation-triggered Na^+^ current was measured when the conditioning pulse was set at −180 mV. No significant difference was detected either in peak Na^+^ current amplitude (−274 ± 50 pA vs. −315 ± 64 pA in control, *P* = 0.3) or voltage-dependent inactivation (half inactivation, *V*_0.5_ is −44 ± 3 mV vs. −40 ± 6 mV in control, *P* = 0.18) between control (*n* = 4) and gluACC1KO (*n* = 6) alpha cells.

Next, we used the sulfonylurea drug tolbutamide to pharmacologically close K_ATP_ channels irrespective of prevailing glucose concentrations. Tolbutamide completely blocked the increase in glucagon secretion at 1 mmol/l glucose in control islets (Fig. 4e). In gluACC1KO islets, tolbutamide exerted no inhibitory effect on glucagon secretion beyond that produced by ablating the ACC1 gene itself (Fig. 4e), consistent with the reduced K_ATP_ channel conductance data (Fig. 4c). Together, these data suggest that reduced K_ATP_ channel activity causes the secretory defect in alpha-cells with genetic or pharmacological inhibition of ACC1.

### Calcium influx is impaired in gluACC1KO alpha cells

**Fig. 5:**
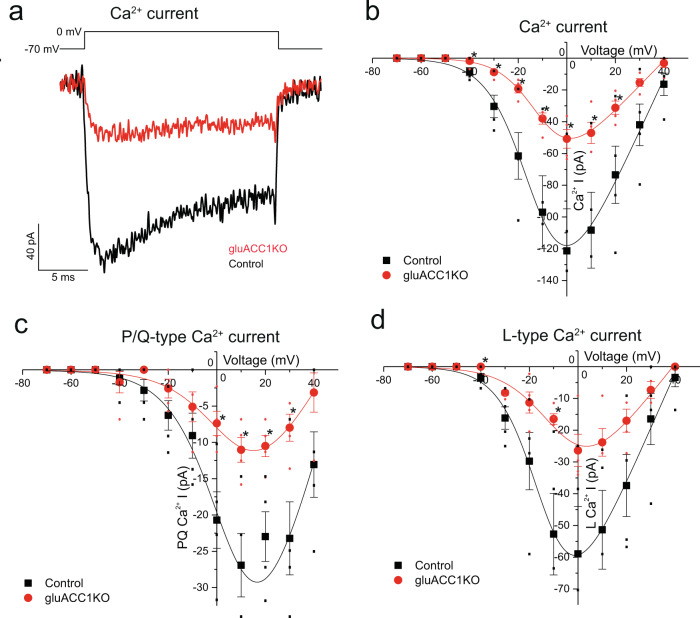
ACC1 is necessary for the maintenance of voltage-gated calcium currents in alpha cells. Electrophysiological recordings of Ca^2+^ currents triggered by a series of 20 ms depolarising pulses (from −70 to +40 mV with a 10 mV step increment) from a holding potential of −70 mV in control (black trace/squares) and gluACC1KO (red trace/circles) alpha cells within intact islets. Current–voltage relationships were fitted with a Boltzmann function. **a**, **b** Representative Ca^2+^ current trace (**a**) and summary of the amplitude of Ca^2+^ currents (**b**) in the presence of 0.1 µg/ml tetrodotoxin to block Na^+^ channels. **c** Summary of the amplitude of P/Q-type Ca^2+^ currents (ω-agatoxin VIA-sensitive component). **d** Summary of the amplitude of L-type Ca^2+^ currents (isradipine-sensitive component). Data from *n* = 4 independent islet preparations analysed by *t*-test. All data presented as mean ± SEM. Significance threshold: *P < 0.05.

We next used high concentrations of K^+^ to depolarise alpha-cells, thereby bypassing K_ATP_ channel-dependent regulation of membrane potential. High-K^+^ treatment increased glucagon secretion in both control and gluACC1KO islets across all glucose concentrations tested, although glucagon release at 1 mmol/l in gluACC1KO islets remained significantly lower than controls (Fig. 4f). These data suggest that loss of ACC1 activity in alpha-cells may cause additional defects in glucagon secretion, distal to K_ATP_ channel activity. To explore this possibility we measured alpha-cell voltage-gated Ca^2+^ currents in gluACC1KO islets. Depolarisation-evoked Ca^2+^ currents in gluACC1KO alpha-cells were significantly smaller (<50%) than in control alpha-cells (Fig. 5a, b). Mouse alpha-cells are equipped with both P/Q- and L-type Ca^2+^ (Ca_V_) channels. The relative contributions of these channel types were assessed using selective antagonists. This analysis revealed that both agatoxin-sensitive P/Q-type Ca^2+^ currents (Fig. 5c) and isradipine-sensitive L-type Ca^2+^ currents (Fig. 5d) were significantly reduced in gluACC1KO alpha-cells relative to controls. Taken together, these data indicate that ACC1 is necessary to maintain Ca_V_ channel functionality in alpha-cells.

### ACC1 regulates alpha cell size and glucagon content

**Fig. 6:**
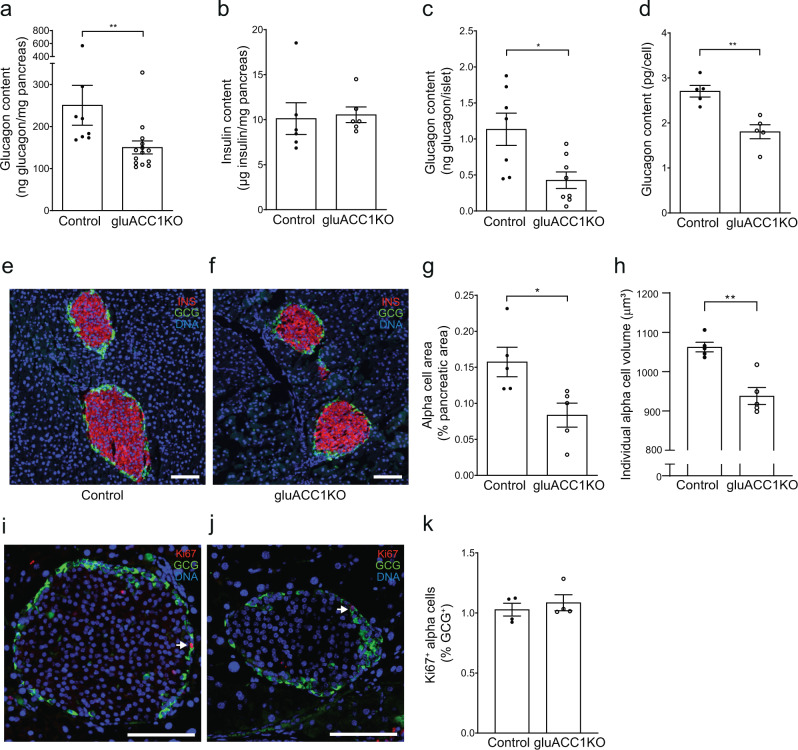
Reduced glucagon content and alpha-cell size in gluACC1KO mice. **a** Pancreatic glucagon content was measured in control (black circles) and gluACC1KO (white circles) adult mice and normalised to pancreatic weight. Data from *n* = 8–14 mice. **b** Pancreatic insulin content measured as in (**a**); *n* = 6 mice. **c** Islet glucagon content measured in batches of 10 size-matched islets, isolated from *n* = 7–8 mice. **d** Glucagon content per cell quantified following fluorescent-activated cell sorting of alpha-cells (Supplementary Fig. 1); *n* = 5 mice. **e**–**h** Formalin-fixed paraffin-embedded pancreata from female mice were sectioned and stained for glucagon, insulin and DAPI before imaging and morphometric analysis using Halo software. **e**, **f** Representative images of pancreatic sections; glucagon (green), insulin (red) and DAPI (blue); scale bar 100 microns. **g** Alpha-cell proportional area: percentage of two-dimensional pancreatic area stained positive for glucagon; *n* = 5 mice. **h** Mean individual alpha-cell volume calculated by dividing the glucagon positive area by the number of nuclei in that area and extrapolating to three dimensions; *n* = 5 mice. **i**–**k** Quantification of proliferating alpha cells. Formalin-fixed paraffin-embedded pancreata from female mice were sectioned and stained for glucagon, Ki67 and DAPI before imaging and cell analysis using Halo software. **i**, **j** Representative images of pancreatic sections; glucagon (green), Ki67 (red) and DAPI (blue); scale bar 100 microns. **k** Quantification of glucagon positive, Kir67 positive cells expressed as a percentage of total glucagon positive cells. *n* = 5 mice. Data in **a**–**d**, **g**, **h** and **k** are presented as mean ± SEM and analysed by Mann–Whitney *U* test. Significance thresholds: *P < 0.05, **P < 0.01.

Given the persistent defect in glucagon secretion in maximally stimulated gluACC1KO alpha-cells in vivo (during ITT, Fig. 2i) and ex vivo (in islets exposed to high-K^+^, Fig. 4f), we considered if a reduction in alpha-cell mass or glucagon content may be present. Pancreatic glucagon content was reduced in gluACC1KO mice (Fig. 6a), whereas insulin content was unaltered (Fig. 6b), reflecting the alpha-cell-specific model. Further analysis revealed that both islet and individual alpha-cell glucagon content (Fig. 6c, d) were reduced, suggesting a loss of glucagon levels and/or a reduction in alpha-cell size. This was quantified by morphometric analysis of immunofluorescent-stained pancreatic sections. Loss of ACC1 did not alter islet organisation: a clear mantle of alpha-cells surrounding a beta-cell core was present in both control and gluACC1KO pancreatic sections (Fig. 6e, f). However, there was a substantial reduction in the total alpha-cell area of the gluACC1KO pancreas (Fig. 6g). Furthermore, we identified a significant reduction in individual alpha-cell size by ~12% in gluACC1KO mice (Fig. 6h), which links the ACC1-coupled pathway with alpha-cell growth. Alpha cell proliferation rates were equivalent between genotypes (Fig. 6i–k), indicating that changes in proliferation do not underpin the alterations in alpha cell mass. As the reduction in cell size (−12%; Fig. 6h) was considerably less than the marked reduction in pancreatic alpha-cell area (−47%; Fig. 6g), this indicates a reduction in both the size and number of alpha-cells. As short-term inhibition of ACC1 in vitro (Fig. 1a–e) phenocopied the impairment of glucagon secretion seen in gluACC1KO islets (Fig. 1h, i), this suggests that the loss of glucagon content and alpha-cell size/number in gluACC1KO mice occurs either independently of, or secondary to, the primary glucose-sensing defect.

### S-acylation of Kir6.2 is regulated by ACC activity

**Fig. 7:**
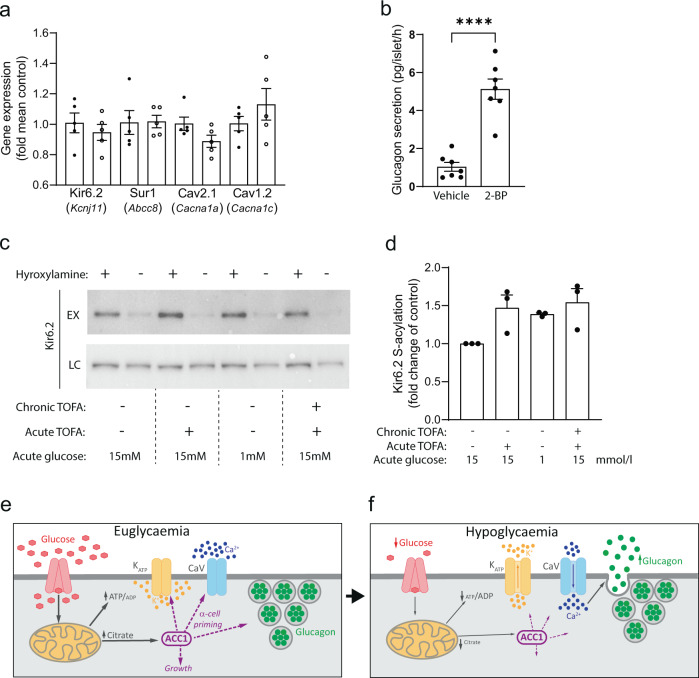
The KATP channel subunit Kir6.2 is S-acylated in alpha cells. **a** mRNA expression of KATP and CaV channel subunits, assessed by RT-PCR using cDNA from FACS-enriched primary alpha cells from control (black circles) and gluACC1KO (white circles) mice. **b** Pancreatic islets isolated from wild-type C57BL6/J mice were pre-treated with vehicle or 100 µmol/l 2-bromopalmitate (2-BP) for 1 h to block protein palmitoylation, before glucagon secretion assays were run. Data from *n* = 7 independent islet preparations analysed by t-test. ****P < 0.0001. **c**-**d** aTC9 cells were cultured for 22 h with 15 mM glucose and either vehicle or 25 mmol/l TOFA (Chronic TOFA), followed by a subsequent 2 h incubation with 1 mM or 15 mM glucose, with vehicle or TOFA (Acute TOFA), before lysis. S-acylated proteins were captured in a hydroxylamine-dependent manner using acyl-biotin exchange (ABE) chemistry. Western blotting for Kir6.2 was performed using ABE eluted samples (EX), with input protein lysates as a loading control (LC). **c** Representative western blot from one experiment. **d** Quantification of western blotting band intensity from *n* = 3 independent experiments. Each EX + band was normalised to the respective LC + band. Data presented as fold-change (with mean ± SEM) and analysed by Mann–Whitney *U* test. **e**, **f** Proposed model for the role of ACC1-coupled signalling in alpha-cell function. During euglycaemia (**e**), ACC1 acts to prime the alpha-cells for secretion, maintaining KATP and CaV channel competency, as well as adequate alpha-cell growth and glucagon content. During a hypoglycaemic stimulus (**f**) glucagon secretion is triggered. In alpha-cells lacking ACC1 activity, glucagon secretion in response to low-glucose concentrations is impaired due to defects in KATP and CaV channel activity, alpha-cell size/number and glucagon content.

As no significant changes in the expression of the K_ATP_ channel (Kir6.2, Sur1) and Cav channel (Cav2.1, Cav1.2) subunits were detected in primary alpha cells from gluACC1KO mice (Fig. 7a), we considered how the loss of ACC1 activity could lead to impaired K_ATP_ channel activity at low glucose in alpha-cells. S-acylation of the K_ATP_ channel subunit, Kir6.2, has recently been reported to impact channel activity in cardiomyocytes^32^. As ACC1 activity influences cellular lipid levels, we reasoned that this metabolic signal may regulate Kir6.2 S-acylation and therefore K_ATP_ channel activity and glucagon secretion in alpha-cells. We first sought to investigate the role of global S-acylation in glucagon secretion in primary islets using 2-bromopalmitate (2-BP) to block S-acylation^33^. Acute 2-BP treatment markedly enhanced glucagon secretion at 1 mM glucose (Fig. 7b), suggesting that S-acylation has a net restraining effect on glucagon secretion. Next, we undertook acyl-biotin exchange (ABE) assays and western blotting which provided clear evidence for Kir6.2 S-acylation in alpha cells (Fig. 7c, d). Interestingly, there was a consistent, albeit non-significant, increase in Kir6.2 S-acylation with either TOFA treatment or 1 mmol/l glucose, suggesting that ACC activity inversely regulates Kir6.2 S-acylation. Moreover, as TOFA treatment impairs K_ATP_ channel activity (Fig. 4d) and glucagon secretion (Fig. 1a–e), our data implicate increased Kir6.2 S-acylation (Fig. 7c, d) in this process. This is consistent with the increase in glucagon secretion induced by disruption of S-acylation with 2-BP (Fig. 7b).

## Discussion

In this study, we present in vivo and in vitro data demonstrating a critical role for ACC1-coupled metabolic signalling in glucagon and GLP-1 secretion. Our use of both primary islets and transformed αTC9 alpha-cells, with ACC1 disrupted using chronic tissue-specific gene knockout approaches or acute small-molecule inhibition, provides clear and consistent evidence for the intrinsic role played by ACC1 in alpha-cell function: in all models, loss of ACC1 activity prevents the increase in glucagon secretion triggered by low glucose. We provide further mechanistic insights, by identifying that K_ATP_ channel conductance at low glucose is impaired in primary gluACC1KO or TOFA-treated alpha cells, explaining why these cells do not respond to hypoglycaemia.

The defect in K_ATP_ conductance was accompanied by a profound reduction of voltage-gated Ca^2+^ (Ca_V_) currents, including P/Q- and L-type components, at experimentally defined membrane potentials corresponding to the action potential peak at low glucose (0–10 mV)^1^, consistent with defective glucagon secretion in gluACC1KO alpha-cells experimentally triggered by high-K^+^. These data are also consistent with a prior study demonstrating that inhibition of fatty acid synthase (the enzyme that utilises malonyl-CoA produced by ACC1 to generate palmitate) reduced calcium currents in bovine chromaffin cells^34^ which, like alpha-cells, possess P/Q-channel activity.

Alongside the pronounced defect in glucagon secretion at low glucose in gluACC1KO islets, we observed a marked reduction in glucagon content of the pancreas. This in part reflected a reduction in both alpha cell proportional area of the pancreas (overall alpha cell mass) and individual alpha cell size. Notwithstanding the ~12% reduction in alpha cell size, individual alpha cell glucagon content was reduced by ~33%, representing a genuine loss of glucagon content, relative to cell size. Therefore, ACC1 activity is required to sustain alpha cell growth and glucagon production. Due to these effects, islet glucagon secretion data were not normalised to glucagon content, as this would inflate apparent secretory rates: instead, these metrics were presented separately. One area for future investigation will be the relationship between ACC1 activity, lipogenesis and mTORC1 signalling: our prior data implicated ACC1 activity in regulating levels of key mTORC1 substrates in beta cells^15^, and a recent study of mice with loss of Raptor in alpha cells demonstrated the essential role of mTORC1 signalling in maintaining glucagon content and alpha cell mass^35^.

Our data using 2-BP treatment demonstrates that protein S-acylation plays a role in glucagon secretion. Moreover, we identify that Kir6.2 is S-acylated in alpha cells, that this is dynamically regulated by glucose, and that this is inversely proportional to ACC activity, suggesting a mechanistic link between ACC-dependent metabolic signalling and ion channel function. An important aspect of these data is the rapidity of the effect: both the enhancement of Kir6.2 S-acylation by TOFA or 1 mmol/l glucose, and the augmentation of glucagon secretion by the S-acylation disruptor 2-BP, occurred with 2 h of treatment. This is consistent with reports that S-acylation/de-acylation can be a rapid signalling event, as for the Src kinase Lck^36^. Future studies will be required to investigate how these post-translational modifications relate to the biophysics of channel gating and glucagon secretion, as well as exploring other proteins that may be modified in alpha cells. For example, regulation of Ca_V_ beta subunit activity and localisation by S-acylation has been reported in other cell types^37,38^.

Whilst our study focussed on pancreatic alpha cell function, we have also uncovered a role for ACC1 in GLP-1 secretion from enteroendocrine cells. Our collective understanding of L-cell function is still developing, with the secretion of GLP-1 known to be dependent on glucose metabolism and K_ATP_ channel regulation^27,39^. This present study implicates anaplerotic mitochondrial metabolism and ACC1-coupled lipogenesis in GLP-1 secretion and suggests that further research into this metabolic pathway in L-cell function is warranted.

An important question relating to this study is whether there are associations between the ACC1 gene and diabetes. Variants in *ACACA* have not previously been associated with type 1 diabetes. However, in the largest type 2 diabetes, GWAS to date^40^, variants within *ACACA* showed a suggestive association (lead variant: rs7211305 *P* = 2.1 × 10^−7^). The available eQTL in the pancreas (GTEx V8, rs62070073, *P* = 7.2 × 10^−6^) was of modest strength and thus we could not determine whether the effect on type 2 diabetes is driven by changes in gene expression. With the increasing power of genetics studies, these suggestive associations will be more robustly tested.

In summary, this study demonstrates that ACC1-coupled metabolic signalling plays a key role in alpha-cell glucose sensing and glucagon secretion, as well as GLP-1 secretion from intestinal L-cells, providing mechanistic insights into the regulation of secretion of these key hormones responsible for systemic metabolic homoeostasis. As hyperglucagonaemia is associated with T2D^3–5^ and other metabolic disorders^41^, our study raises the prospect of targeting the ACC1-pathway in alpha-cells as a therapeutic strategy to limit glucagon secretion.

## Methods

### Gene expression and western blotting

RNA from snap-frozen tissues was isolated using RNeasy Mini Kits (QIAGEN, Hilden, Germany) following mechanical homogenisation. Reverse transcription was performed using random primers. Expression was measured by Real-Time PCR using Taqman probes for ACC1 (m*Acaca* Mm01304281_m1), ACC2 (m*Acacb* Mm01204671_m1) and HPRT1 (mHtrp Mm00446968_m1) as a normalising gene. For western blotting, snap-frozen tissues or cell pellets were lysed in RIPA buffer with mechanical homogenisation, and 25 µg protein resolved by sodium dodecyl sulfate-polyacrylamide gel electrophoresis (SDS-PAGE) (10% Acrylamide), before transfer to PVDF, blocking and incubation with anti-ACC1 (#4190; Cell Signalling Technologies, Danvers, MA, USA; 1/1000 dilution) then an HRP-conjugated secondary antibody (Cell Signalling Technologies; 1/10,000 dilution).

### Mouse models

Ethical approval for studies involving mice was provided by the UK Home Office to Dr. J Cantley (project license P4D4BEF1F) following local ethical review (University of Oxford). To disrupt ACC1 activity in proglucagon expressing cells, *Acaca* floxed mice^23^ (mice with loxP sites flanking exons 42 and 43 of the *Acaca* gene) were crossed with glucagon-Cre mice^22^ (Gcg^tm1.1(icre)Gkg^) to generate compound heterozygotes (Gcg^tm1.1(icre)Gkg^, *Acaca*^flox/wt^), which were crossed to generate gluACC1KO (Gcg^tm1.1(icre)Gkg^, *Acaca*^flox/flox^) and control (*Acaca*^flox/flox^) mice. Control and gluACC1KO mice were crossed to generate study cohorts which consisted of gluACC1KO and littermate ACC1-floxed control mice. Mice were maintained on a C57Bl/6J background with a 12 h light/dark cycle, free access to water and a standard chow or HFHS diet (D12451; Research Diets, NJ, USA). For experiments using wild type tissue, C57Bl/6J mice were used. Routine genotyping was performed using primers for ACC1 (ACC1 F: TACAAACGCAAGAGTCATACTGG, ACC1 R: CTTTCCAATTCAAGGTTCTGAC) and for improved Cre (iCre F: GACAGGCAGGCCTTCTCTGAA, iCre R: CTTCTCCACACCAGCTGTGGA). Confirmation of deletion of the ACC1 floxed allele was performed by PCR^15^, following genomic DNA extraction from snap-frozen tissues using the QIAamp Fast DNA Tissue Kit (Qiagen), and using the primers ACC1F (TACAAACGCAAGAGTCATACTGG) and ACC1KOR (CACACCAGTATTTGAATCAGCAA). The primers MIR-F (CAAATGTTGCTTGTCTGGTG) and MIR-R (GTCAGTCGAGTGCACAGTTT) were used as a template control.

### Metabolic phenotyping

Glucose tolerance tests were performed after a 6 h fast, by i.p. administration of glucose 2 g/kg body weight (IPGTT). Insulin action was assessed, following a 6 h fast, by i.p. injection of insulin: 0.75 U/kg for males and 0.5 U/kg for females^42^. Blood was sampled from the tail. Glucose was measured from whole blood using an Accu-Check glucometer (Roche, Basel, Switzerland). Serum glucagon was assayed by ELISA (Mercodia, Uppsala, Sweden). Serum insulin was assayed by MSD immunoassay (Meso Scale Discovery, Rockville, MD, USA). Total serum GLP-1 was assayed by ELISA (Sigma-Aldrich, St. Louis, MO, USA).

### Islet and cell line experiments

Pancreatic islets were isolated^15^ by perfusion of the pancreatic ductal system with collagenase and thermolysin (Liberase, Roche) before digestion at 37 °C, mechanical disruption and islet separation using a Ficoll-Paque plus gradient (GE Healthcare, Chalfont St Giles, UK)^15^. Ex vivo glucagon secretion experiments were performed on the same day as islet isolation using KRBH^15^ supplemented with 0.1% bovine serum albumin: islets were pre-incubated for 1 h with 6 mmol/l glucose prior to incubation of batches of islets for 1 h in the presence of the indicated glucose concentrations to assess secretion. Dynamic glucagon secretion was assessed using perifusion apparatus (Biorep, Miami Lakes, FL, USA) with a flow rate of 25 µl/min and 3 min collection fractions. AlphaTC1 clone 9 (αTC9) cells^20,21^ were a kind gift from Dr. Catriona Kelly (University of Ulster). Cells were maintained in DMEM supplemented with 15 mmol/l glucose, 15 mmol/l Hepes, 10% fetal calf serum and 1% penicillin/streptomycin. The glucose concentration was reduced to 2.5 mmol/l for 16 h prior to experiments. During pharmacological experiments, compound or vehicle was added to either the overnight culture media (chronic/24 h treatment) or to the pre-incubation and stimulation buffer (acute/1 h treatment). Secreted insulin and glucagon were quantified by time-resolved Förster resonance energy transfer (TR-FRET) immunoassay (Cisbio/Perkin Elmer, Waltham, MA, USA). Gut crypts were isolated as described^43^ and incubated overnight before secretion experiments were conducted. Glutag cells^44^ were a kind gift from Prof. Daniel J. Drucker (Mount Sinai Hospital, Toronto) and were maintained on 0.4% Matrigel (Corning, NY, USA). GLP-1 secretion experiments were performed for 2 h, following a 1 h pre-incubation in KRBH containing 0.5 mmol/l glucose for 1 h. Active GLP1 in the supernatant was quantified by TR-FRET immunoassay (Cisbio/Perkin Elmer).

### Electrophysiological recordings

All electrophysiological measurements were performed using an EPC-10 patch–clamp amplifier and Pulse software (version 8.80, HEKA Electronics, Lambrecht/Pfalz, Germany). K_ATP_ channel activity was monitored in alpha cells within intact islets using the perforated patch-clamping technique^45^. In brief, whole-cell K^+^ conductance (reflecting K_ATP_ channel conductance) was estimated by measurement of currents trigged by ±10 mV excursions (200 ms) from −70 mV in voltage-clamped alpha-cells. The extracellular solution contained (in mmol/l): 140 NaCl, 3.6 KCl, 0.5 MgCl_2_, 1.5 CaCl_2_, 10 HEPES, 0.5 NaH_2_PO_4_, NaHCO_3_ and glucose as indicated (pH = 7.4 by NaOH). The pipette solution contained (in mmol/l): 76 K_2_SO_4_, 10 NaCl, 10 KCl, 1 MgCl_2_ and 5 HEPES (pH = 7.35 by KOH). Membrane-perforating amphotericin B was added to the pipette solution (24 µg/ml final concentration) to establish electrical contact with the cell interior.

Transmembrane currents were recorded from superficial alpha-cells in intact, freshly isolated mouse pancreatic islets from control and gluACC1KO mice, using the standard whole-cell configuration. The extracellular solution contained (in mmol/l): 118 NaCl, 20 Tetraethylammonium chloride (TEA-Cl), 5.6 KCl, 1.2 MgCl_2_, 5 HEPES, 2.6 CaCl_2_ and 5 d-glucose (pH 7.4 with NaOH). The pipette solution contained (in mmol/l): 120 CsCl, 1 MgCl_2_·6H_2_O, 1 CaCl_2_, 10 EGTA, 10 Hepes and 3 Mg-ATP (pH 7.15 with CsOH).

Sodium and calcium currents were extracted from whole-cell current recordings and used to plot current–voltage (IV) relationships and voltage-dependent inactivation of sodium currents. Depolarisation-triggered calcium currents were isolated using a broad-spectrum voltage-gated sodium channel blocker tetrodotoxin (TTX, Alomone Labs, Jerusalem, Israel) which was used at a final concentration of 0.1 μg/ml in the extracellular solution. The IV relationships were fitted with a Boltzmann IV function and the voltage-dependent sodium current inactivation was fitted with a single Boltzmann function, using the non-linear curve fitting feature of OriginPro 2017 (OriginLab, Northampton, MA, USA). Alpha cells were identified by immunocytochemistry post electrophysiological recordings^46^. Briefly, biocytin was injected via the patch pipettes during electrophysiological data acquisition to label the recorded cells. Islets were then collected and fixed in 4% paraformaldehyde prior to immunostaining for glucagon, insulin and/or somatostatin. Biocytin was stained using a fluorophore-conjugated streptavidin (Alexa Fluor 568 Streptavidin, Thermo Fisher).

### Hormone content

Total glucagon and insulin content was quantified from the whole pancreas or batches of 10 islets homogenised in ice-cold acid–ethanol (75% ethanol, 1.5% HCl). Total active GLP-1 content was quantified from intestines homogenised in ice-cold acid ethanol. Hormone content was assayed as described for islet experiments.

### Histology

Formalin-fixed paraffin-embedded pancreases were sectioned and immunostained using anti-glucagon (G2654; Sigma-Aldrich; 1/250 dilution), anti-insulin (A0564; Dako, Glostrup, Denmark; 1/250 dilution) and anti-Ki67 (ab15580; Abcam, Cambridge, UK; 1/250 dilution), with Alexa fluor-conjugated secondary antibodies (Molecular Probes/Thermo Fisher, Waltham, MA, USA; 1/250 dilution). For analysis of alpha-cell size, number and proliferation, stained sections were imaged with an Olympus vs120 SW slide scanner (Olympus, Tokyo, Japan) and analysis was performed using Halo software (Indica Labs, Albuquerque, New Mexico USA): alpha-cell mass and number were calculated using data averaged from four pancreatic sections per mouse.

### Fluorescence-activated cell sorting

Isolated islets (as above) were handpicked, washed and dissociated in 0.25% Trypsin EDTA (ThermoFisher) for 10 min at 37 °C with agitation. Dissociated cells were washed and filtered (70 μM) and putative alpha and beta-cells (live singlets) sorted based on forward and side scatter patterns^47^, using a FACSAria II cell sorter (Becton Dickinson, Eysins, Switzerland).

### S-acylation assays

S-acylation assays used ABE chemistry and were performed as described^48^. In brief, αTC9 cells were lysed in LB (100 mM Tris.HCl pH 7.2, 5 mM EDTA, 2.5% SDS) containing 10 mM N-ethylmaleimide to block free cysteines. Chromosomal DNA was sheared by passage through a 25 gauge needle and insoluble debris pelleted at 16K × g for 10 min. A 500 μg total protein (quantified using the BCA method) was diluted to 1.1 ml in LB + 10 mM N-ethylmaleimide and incubated at room temperature for one hour with mixing. N-ethylmaleimide was removed by reaction with 2,3-dimethyl-1,3-butadiene at room temperature with vigorous shaking for 1 h. Excess 2,3-dimethyl-1,3-butadiene and reacted N-ethylmaleimide was extracted by addition of 100 μl chloroform, vigorous mixing and phase partitioning by centrifugation. A 500 μl of the upper aqueous phase was mixed with 500 μl 1 M hydroxylamine pH 7.2 and 100 μl 1 mM biotin-HPDP (Thermo Fisher) in DMSO and incubated for 1 h at room temperature with mixing. The remaining 500 μl of the upper phase was treated identically except that 1 M NaCl was substituted for hydroxylamine. Samples were chloroform/methanol precipitated, air-dried and resuspended in 200 μl 100 mM Tris.HCl pH 7.2, 1 mM EDTA, 2% SDS, 8 M urea with gentle mixing. 20 ul was retained as a loading control and mixed with 20 μl 2× reducing SDS-PAGE sample buffer. The remaining sample was diluted to 1.4 ml with PBS and 40 ul of 50% high capacity neutravidin beads (Thermo Fisher) added and mixed at room temperature for 2 h to capture biotinylated (previously S-acylated) proteins. Beads were washed 3× with PBS containing 1% SDS, and twice with PBS, before elution in 2× reducing SDS-PAGE sample buffer at 37 °C for 30 min with agitation. Protein S-acylation state was determined by SDS-PAGE and western blotting using an anti-Kir6.2 (Kcnj11) primary antibody (Santa Cruz Biotechnology Inc., Dallas, TX, USA; 1/1000 dilution) and an HRP-conjugated secondary antibody (Cell Signalling Technologies; 1/5000 dilution). Western blot signal was captured using super signal west pico ECL substrate (Thermo Fisher) and a G:BOX XT4 imager (Syngene, Cambridge, UK). Image data were analysed and quantified using GeneTools software (Syngene).

### Statistics and reproducibility

Data are presented as mean ± SEM with individual data points shown. Samples sizes and definitions of replicates are reported in the figure legends. Simple pairwise comparisons were tested using two-tailed *t*-tests or Mann–Whitney tests (for non-parametric data): tests were unpaired unless otherwise indicated. Multiple comparisons were made using one-way ANOVA with Tukey’s post-test, or two-way ANOVA with Fisher’s LSD test, as indicated. A *P* value < 0.05 was regarded as statistically significant. Statistical analysis was performed using Prism8 (Graphpad Software, San Diego, CA, USA).

### Chemicals

All reagents were purchased from ThermoFisher (Waltham, MA, USA) unless otherwise stated. TOFA (5-(tetradecyloxy)-2-furoic acid^13^; ab141578) and Tolbutamide were purchased from Abcam; Noradrenaline from Tocris Bioscience (Bristol, UK); 2-Bromopalmitate from Sigma-Aldrich; Tetrodotoxin, ω-agatoxin and isradipine from Alomone Labs (Jerusalem, Israel).

### Reporting summary

Further information on research design is available in the Nature Research Reporting Summary linked to this article.

## Supplementary information

Supplementary Information

Description of Additional Supplementary Files

Supplementary Data 1

Reporting Summary

## Data Availability

Source data are provided as a supplementary data file (Supplementary Data 1). Uncropped western blots are provided in the supplementary information (Supplementary Fig. 6). All other data are available from the corresponding author on reasonable request.
