# Supplementary material for: Acetyl-CoA-carboxylase 1 (ACC1) plays a critical role in glucagon secretion
Source: Commun Biol. 2022 Mar 18;5:238. doi: 10.1038/s42003-022-03170-w (PMC8933412; doi:10.1038/s42003-022-03170-w)
Supplement: Supplementary file 1 — Supplementary Information [file 42003_2022_3170_MOESM1_ESM.pdf]

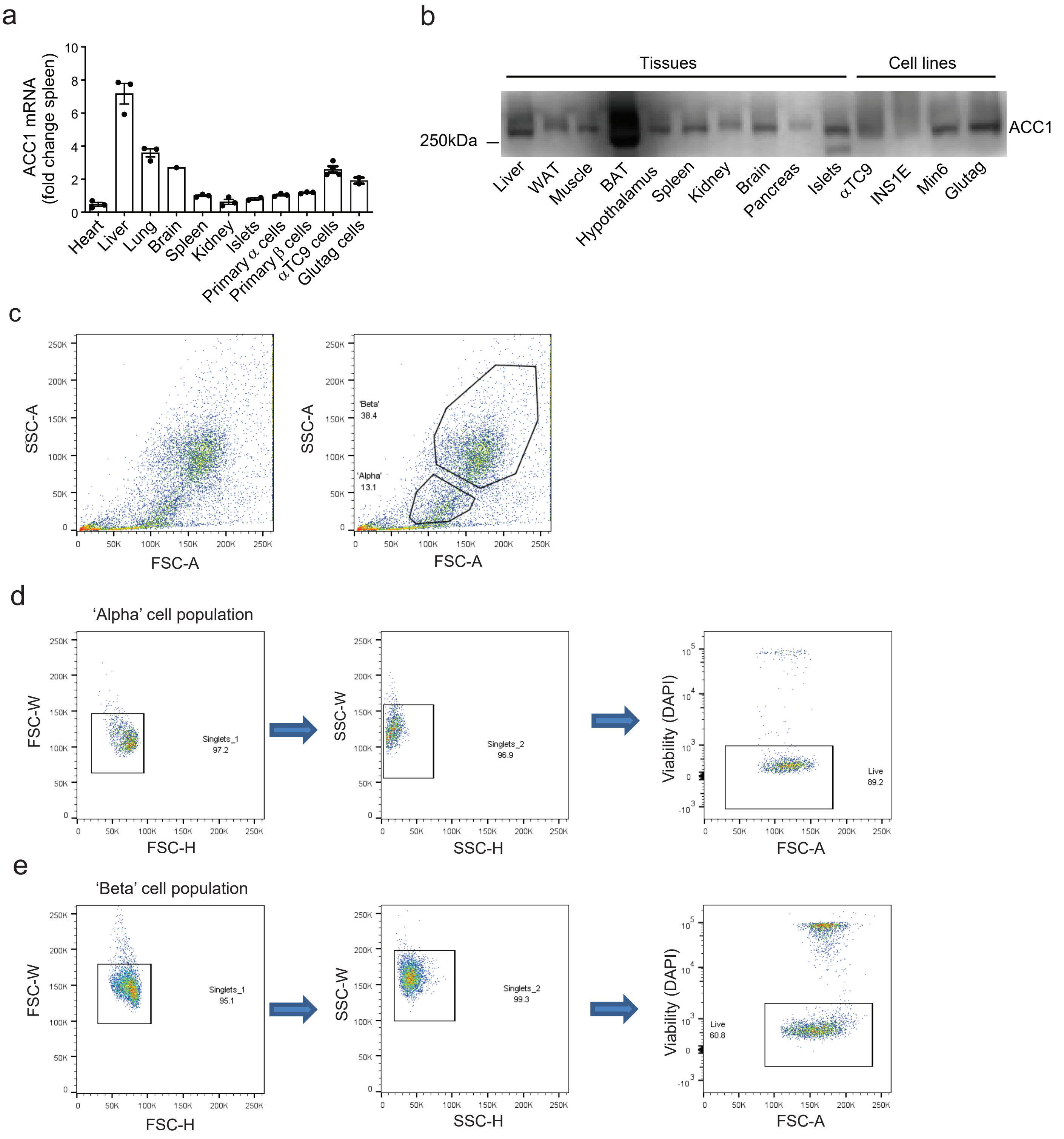

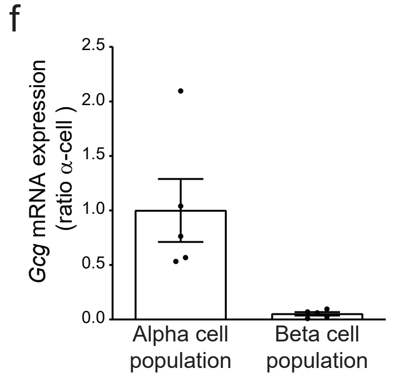

Supplementary figure 1: Comparative ACC1 expression across tissue types a. ACC1 (*Acaca*) mRNA expression was measured in tissues from wild-type C57BL6/J mice by RT-PCR, with results normalised to *Hprt1* and presented relative to expression in the spleen. Data presented as mean±SEM; n=1 for brain, n=2-4 for all other tissues. **b**. ACC1 protein levels from the indicated tissues assessed by western blotting. **c-e**. Fluorescence-activated cell sorting (FACS) enrichment of alpha and beta-cell populations from dissociated islets, based on forward and side scatter properties, using a FACSAria II cell sorter (Becton Dickinson). Cell viability was determined by DAPI exclusion. **f**. Glucagon mRNA expression in alpha and beta populations post-FACS**.** Data from n=5 individual islet/cell preparations.

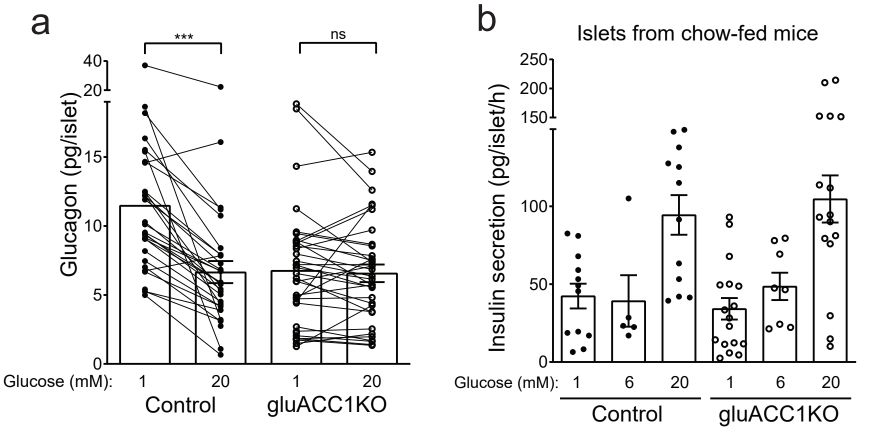

Supplementary figure 2: Genetic inactivation of ACC1 impairs glucagon secretion. a. Glucose-regulation of glucagon secretion is lost in gluACC1KO islets. Re-analysis of data presented in Fig.1h with paired measurements: data from n=24-35 independent islet preparations analysed by paired t-test. **b**. Insulin secretion assessed from isolated islets. Data from n=5-17 independent islet preparations. Data presented as mean±SEM. Significance thresholds: ****P*<0.001.

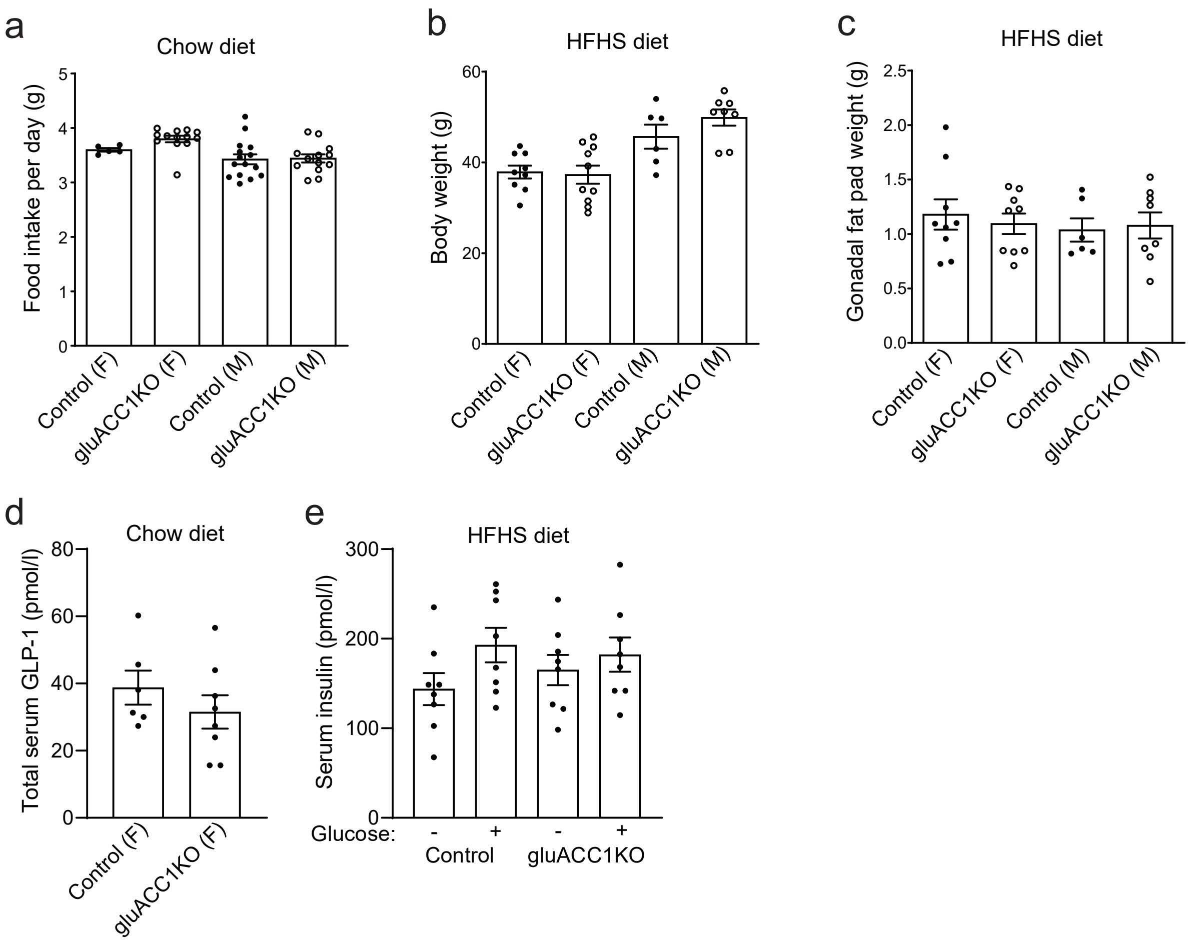

Supplementary figure 3. Food intake, adiposity and serum GLP-1 in gluACC1KO mice.
a. Food intake measured in mice group-housed by genotype for 3 weeks on a standard chow diet (n=5-15 mice). **b**. Body weight following 12 weeks feeding with high-fat high-sucrose (HFHS) diet (n=6-10 mice). **c**. Epidydimal (M) or periovarian (F) fat pads weighed following 12 weeks of feeding with a HFHS diet (n=6-9 mice). **d.** Female mice fasted for 16h received an oral glucose bolus (3g/kg) 5 mins prior to serum collection for total GLP-1 assays (n=6-8 mice). **e**. Female mice following 12 weeks feeding with high-fat high-sucrose (HFHS) diet were fasted for 16 hours and serum insulin collected before and after an oral glucose bolus. Data presented as mean±SEM, for female (F) and male (M) mice as indicated.

a

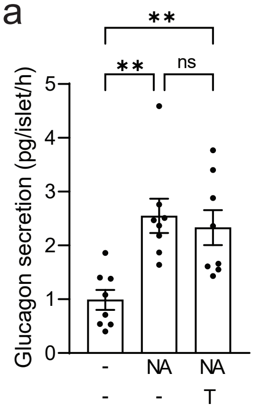

Supplementary figure 4. Effect of adrenaline on glucagon secretion from islets with acute ACC1 inhibition. a. Islets isolated from wild-type C57Bl6/J mice were pre-incubated with 25uM TOFA or vehicle for 60mins, before stimulation with 1mM glucose with addition of vehicle, 5uM noradrenaline (NA) and/or 25uM TOFA (T) for 60mins as indicated. Equal concentration of DMSO vehicle was present in all conditions. Data from n=8 independent islet preparations, presented as mean±SEM and analysed by one-way ANOVA with Tukey's multiple comparison test (***P*<0.01; ns = not signficant).

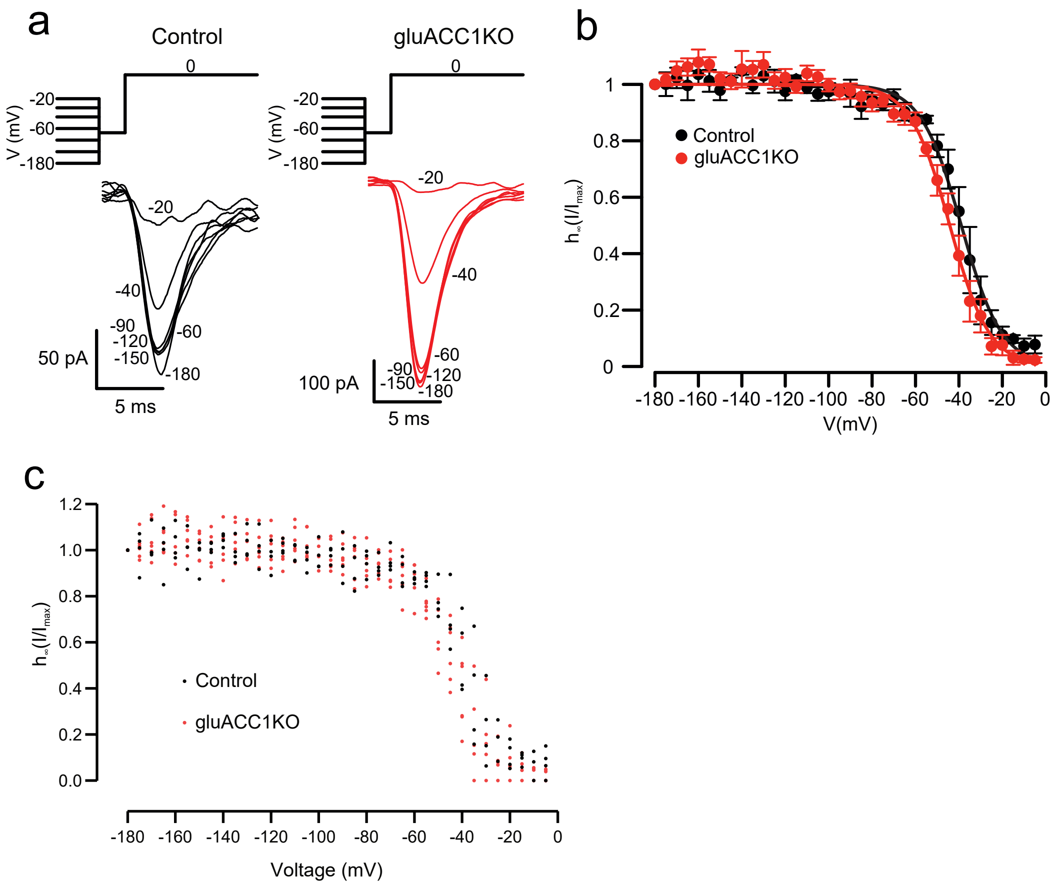

Supplementary Figure 5. Loss of ACC1 does not affect Na$^+$ current properties in alpha cells. a. Na$^+$ currents triggered by test pulses (10 ms, 0 mV) following conditioning pulses (200 ms, voltages as indicated) in alpha cells from control (black) and gluACC1KO (red) mice. **b, c.** Voltage-dependent inactivation of Na$^+$ current in control (black) and gluACC1KO (red) alpha cells. Currents (I) were normalised to peak Na$^+$ currents measured when conditioning pulse was set at -180 mV (Imax). Data are presented as h∞(I/Imax), mean±SEM, with curves representing Boltzmann fits to the data. **c**. Individual data points used to generate plots in panel b.

a

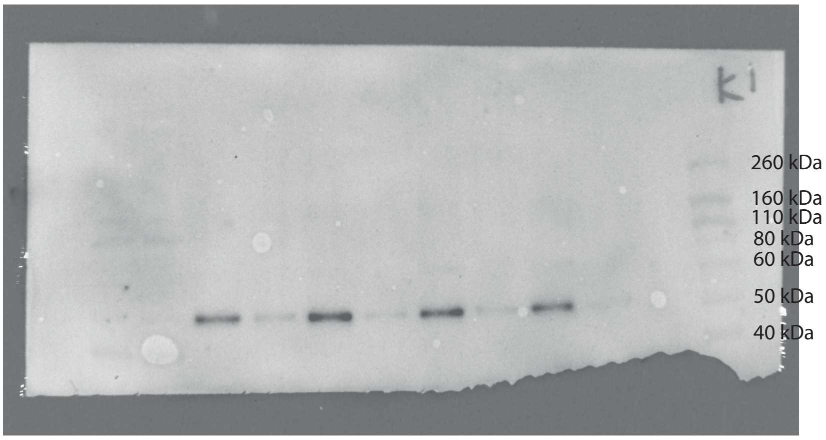

b

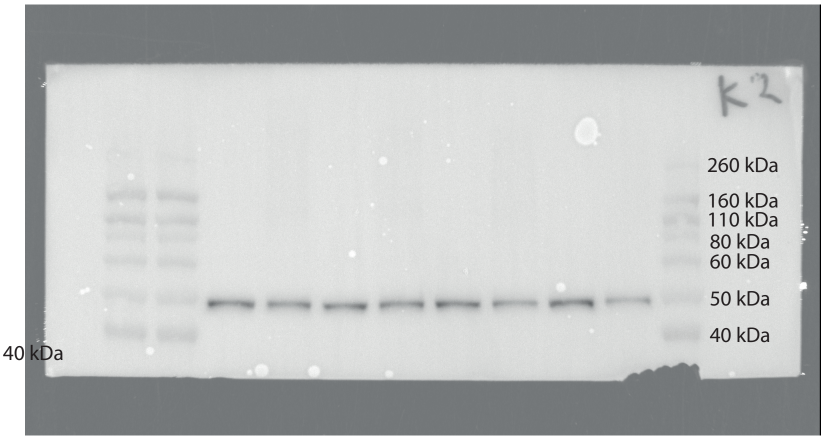

c

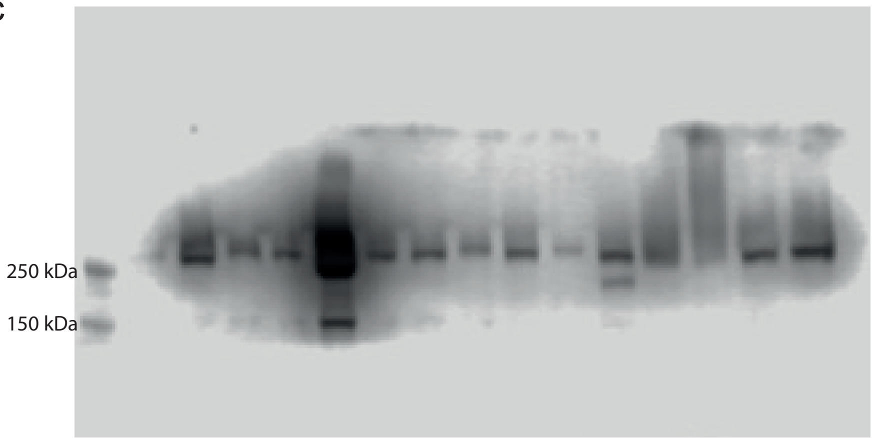

Supplementary Figure 6: Uncropped western blots.

a. Western blot from Fig.7c EX.

b. Western blot from Fig.7c LC.

c. Western blot from supplementary Fig.1b.
