# Supplementary material for: Acetyl-CoA-carboxylase 1 (ACC1) plays a critical role in glucagon secretion
Source: Commun Biol. 2022 Mar 18;5:238. doi: 10.1038/s42003-022-03170-w (PMC8933412; doi:10.1038/s42003-022-03170-w)
Supplement: Supplementary file 2 — Description of Additional Supplementary Files [file 42003_2022_3170_MOESM2_ESM.pdf]

Description of Additional Supplementary Files

File name: Supplementary Data 1
Description: Source data for the graphs/figures.
