# Supplementary material for: Acetyl-CoA-carboxylase 1 (ACC1) plays a critical role in glucagon secretion
Source: Commun Biol. 2022 Mar 18;5:238. doi: 10.1038/s42003-022-03170-w (PMC8933412; doi:10.1038/s42003-022-03170-w)
Supplement: Supplementary file 4 — Reporting Summary [file 42003_2022_3170_MOESM4_ESM.pdf]

nature research

Corresponding author(s): Dr. James Cantley

Last updated by author(s): 2021/12/21

Reporting Summary

Nature Research wishes to improve the reproducibility of the work that we publish. This form provides structure for consistency and transparency in reporting. For further information on Nature Research policies, see our Editorial Policies and the Editorial Policy Checklist.

Statistics

For all statistical analyses, confirm that the following items are present in the figure legend, table legend, main text, or Methods section.

n/a	Confirmed	
☐	☒	The exact sample size (*n*) for each experimental group/condition, given as a discrete number and unit of measurement
☐	☒	A statement on whether measurements were taken from distinct samples or whether the same sample was measured repeatedly
☐	☒	The statistical test(s) used AND whether they are one- or two-sided<br>*Only common tests should be described solely by name; describe more complex techniques in the Methods section.*
☒	☐	A description of all covariates tested
☐	☒	A description of any assumptions or corrections, such as tests of normality and adjustment for multiple comparisons
☐	☒	A full description of the statistical parameters including central tendency (e.g. means) or other basic estimates (e.g. regression coefficient) AND variation (e.g. standard deviation) or associated estimates of uncertainty (e.g. confidence intervals)
☐	☒	For null hypothesis testing, the test statistic (e.g. *F*, *t*, *r*) with confidence intervals, effect sizes, degrees of freedom and *P* value noted<br>*Give P values as exact values whenever suitable.*
☒	☐	For Bayesian analysis, information on the choice of priors and Markov chain Monte Carlo settings
☒	☐	For hierarchical and complex designs, identification of the appropriate level for tests and full reporting of outcomes
☒	☐	Estimates of effect sizes (e.g. Cohen's *d*, Pearson's *r*), indicating how they were calculated

Our web collection on statistics for biologists contains articles on many of the points above.

Software and code

Policy information about availability of computer code

Data collection	All electrophysiological measurements were performed using an EPC-10 patch-clamp amplifier and Pulse software (version 8.80, HEKA Electronics, Lambrecht/Pfalz, Germany).<br>For alpha-cell size and number, stained sections were imaged with an Olympus vs120 SW slide scanner and VS-ASW acquisition software (Olympus, Tokyo, Japan).<br>Hormone concentrations were determined using plate-based immunoassays (ELISAs, TR-FRET) with data acquired using a Spark 10M multi-mode plate reader with SparkControl software (Tecan, Switzerland).<br>FACS was performed using a FACSAria II cell sorter with FACSDiva control software (Becton Dickinson, Eysins, Switzerland).<br>Signal from S-acylation assays was captured using a G:BOX XT4 imager (Syngene, Cambridge, UK).
Data analysis	Statistical analysis and graphing were performed using Prism8 (Graphpad Software, San Diego, CA, USA).<br>Membrane currents collected during electrophysiological recordings were fitted with a Boltzmann IV curve using the non-linear curve fitting feature of OriginPro 2017 (OriginLab, Northampton, MA, USA).<br>For alpha-cell size and number, images of stained sections were analysed using Halo software (Indica Labs, Albuquerque, New Mexico USA).<br>Hormone concentrations were determined using plate-based immunoassays (ELISAs, TR-FRET): data were analysed using Magellan software (Tecan, Switzerland) and GraphPad prism (above).<br>FACS/flow data were analysed using FlowJo software (Becton Dickinson, Eysins, Switzerland).<br>S-acylation data was analysed and quantified using GeneTools software (Syngene, Cambridge, UK).

For manuscripts utilizing custom algorithms or software that are central to the research but not yet described in published literature, software must be made available to editors and reviewers. We strongly encourage code deposition in a community repository (e.g. GitHub). See the Nature Research guidelines for submitting code & software for further information.

Data

Policy information about availability of data

All manuscripts must include a data availability statement. This statement should provide the following information, where applicable:

- Accession codes, unique identifiers, or web links for publicly available datasets
- A list of figures that have associated raw data
- A description of any restrictions on data availability

Source data are provided as a supplementary data file.

Field-specific reporting

Please select the one below that is the best fit for your research. If you are not sure, read the appropriate sections before making your selection.

☒ Life sciences ☐ Behavioural & social sciences ☐ Ecological, evolutionary & environmental sciences

For a reference copy of the document with all sections, see nature.com/documents/nr-reporting-summary-flat.pdf

Life sciences study design

All studies must disclose on these points even when the disclosure is negative.

Sample size	The sample sizes were chosen primarily based on achieving a sufficient number of biological replicates to provide a significant control response for the given technique/assay, against which the effect of genetic/pharmacological inhibition of ACC1 could be tested. For example, in figure 1A we used a sufficient number of biological replicates to achieve a significant change in glucagon secretion between 1mM and 20mM glucose in control (vehicle-treated) cells, which then enabled us to test the effect of inhibiting ACC1 on this response. The final sample size was further influenced by the generation and availability of genetically modified mice (an individual mouse was treated as 1 biological replicate), whereby balanced numbers of mice per group could not always be achieved.
Data exclusions	Data points were only excluded in exceptional circumstances such as relating to a technical issue or artefact. For example, if a mouse did not respond to glucose during a glucose tolerance test (i.e. blood glucose did not change) data from this mouse would be excluded as this indicates an underlying failure in i.p. delivery.
Replication	For mouse and primary cell studies:<br>First, we treated each mouse (or tissues derived from a mouse) as a single biological replicate in our statistical analyses, ensuring that the data presented represent the response of multiple animals.<br>Second, data were collected from multiple cohorts of mice on different experimental days.<br><br>For cell line studies:<br>We present mean data points from independent experiments, and have paired these data points by experimental day across different treatment conditions to clearly show the effect within each experiment and how this related to other experiments (Fig.1d,e).
Randomization	Our breeding strategy ensured that each litter consisted of both control and gluACC1KO mice. At weaning, technicians without any knowledge of the scientific question would number mice: as our gluACC1KO mice were indistinguishable from littermate controls, this ensures that the order in which mice were numbered is essentially a random sequence of control and gluACC1KO mice. These litters (when adults) were then entered into phenotyping pipelines: during a given experiment (e.g. GTT) mice were studied sequentially based on the number allocated at weaning, effectively producing a random sequence of controls and KOs in each cohort/experiment.
Blinding	Whilst it was not possible to undertake strict blinding for most experiments (i.e. the researcher responsible for breeding also conducted the phenotyping), during the conduct of experiments mice/cells were identified by ID number rather than by genotype, minimising any potential for observer bias. For histology studies, images were captured using an automated slide scanner and analysed using automated analysis software, removing the potential for observer bias.

Reporting for specific materials, systems and methods

We require information from authors about some types of materials, experimental systems and methods used in many studies. Here, indicate whether each material, system or method listed is relevant to your study. If you are not sure if a list item applies to your research, read the appropriate section before selecting a response.

Materials & experimental systems

n/a	Involved in the study
☐	☒ Antibodies
☐	☒ Eukaryotic cell lines
☒	☐ Palaeontology and archaeology
☐	☒ Animals and other organisms
☒	☐ Human research participants
☒	☐ Clinical data
☒	☐ Dual use research of concern

Methods

n/a	Involved in the study
☒	☐ ChIP-seq
☐	☒ Flow cytometry
☒	☐ MRI-based neuroimaging

Antibodies

Antibodies used	For western blotting:<br>25 µg protein was resolved by SDS-PAGE (10% Acrylamide), before transfer to PVDF, blocking and incubation with anti-ACC1 (#4190; Cell Signalling Technologies, Danvers, MA, USA; 1/1000 dilution) then an HRP-conjugated secondary antibody (Cell Signalling Technologies; 1/10,000 dilution).<br><br>For histology:<br>Formalin-fixed paraffin-embedded (FFPE) pancreases were sectioned and immunostained using anti-glucagon (G2654; Sigma, St. Louis, MS, USA; 1/250 dilution), anti-insulin (A0564; Dako, Glostrup, Denmark; 1/250 dilution) and Alexafluor-conjugated secondary antibodies (Molecular Probes/Thermo Fisher, Waltham, MA, USA; 1/250 dilution).<br><br>For S-acylation assays:<br>Protein outputs from the S-acylation (ABE) assays were analysed by western blotting (as above) using an anti-Kir6.2 (Kcnj11) antibody (sc-390104, Santa Cruz Biotechnology Inc., Dallas, TX, USA; 1/1000 dilution) and an HRP-conjugated secondary antibody (Cell Signalling Technologies, Danvers, MA, USA; 1/5000 dilution).
Validation	Antibodies were used in accordance with the manufacturers instructions, and using established lab protocols.<br><br>For western blotting: the resulting band was clear and distinct and at the expected molecular weight for ACC1 (269kDa).<br><br>For histology: The pattern of staining for the antibodies used in our histology analysis picks up the distinctive core of beta cells (insulin) and mantle of alpha cells (glucagon) of the mature rodent islet (Fig.6e, f), with minimal signal from exocrine tissue, validating these antibodies.<br><br>For S-acylation assays: The resulting band was clear and distinct and at the expected molecular weight for Kir6.2.

Eukaryotic cell lines

Policy information about cell lines

Cell line source(s)	AlphaTC1 clone 9 (αTC9) cells were a kind gift from Dr. Catriona Kelly (University of Ulster).<br>Glutag cells were a kind gift from Prof. Daniel J. Drucker (Mount Sinai Hospital, Toronto).
Authentication	aTC9 cells demonstrated glucose-regulated glucagon secretion (quantified by immunoassay) thereby demonstrating an alpha cell phenotype.<br>Glutag cells demonstrated GLP1 secretion (quantified by immunoassay) thereby demonstrating an L-cell phenotype.
Mycoplasma contamination	Cell lines were not tested for mycoplasma.
Commonly misidentified lines (See ICLAC register)	The two cell lines used could not be found on the ICLAC register.

Animals and other organisms

Policy information about studies involving animals; ARRIVE guidelines recommended for reporting animal research

Laboratory animals	Mus musculus (mice): strain C57Bl6/J. Genetically modified lines: Gcgtm1.1(icre)Gkg and Acaca flox/flox.
Wild animals	No wild animals were used.
Field-collected samples	The study did not involve field-collected samples.
Ethics oversight	Ethical approval for this work was provided by the UK home office to Dr. Cantley (project license P4D4BEF1F) following local review of the project license and study protocols by the Animal Welfare and Ethical Review Board at the Department of Physiology, Anatomy and Genetics, University of Oxford.

Note that full information on the approval of the study protocol must also be provided in the manuscript.

Flow Cytometry

Plots

Confirm that:

- [x] The axis labels state the marker and fluorochrome used (e.g. CD4-FITC).
- [x] The axis scales are clearly visible. Include numbers along axes only for bottom left plot of group (a 'group' is an analysis of identical markers).
- [x] All plots are contour plots with outliers or pseudocolor plots.
- [x] A numerical value for number of cells or percentage (with statistics) is provided.

Methodology

Sample preparation	Isolated islets (as above) were handpicked, washed and dissociated in 0.25% Trypsin EDTA (ThermoFisher) for 10 min at 37degC with agitation. Dissociated cells were washed and filtered (70 µM) and putative alpha and beta cells (live singlets) sorted based on forward and side scatter patterns.
Instrument	FACSAria II cell sorter (Becton Dickinson, Eysins, Switzerland).
Software	Collection/instrument operation: FACSDiva (Becton Dickinson, Eysins, Switzerland).<br>Analysis: FlowJo (Becton Dickinson, Eysins, Switzerland).
Cell population abundance	'Beta' population 38.4%, 'Alpha' population 13.1%, within cells sorted.
Gating strategy	Supplementary Figure 1 clearly shows the gating strategy, including forward scatter, side scatter and viability (DAPI).

- [x] Tick this box to confirm that a figure exemplifying the gating strategy is provided in the Supplementary Information.
